# The mechanisms of lncBMP4 mediating *BMP4* promoter activity to regulate the proliferation of dermal papilla cells in Hu sheep

**DOI:** 10.5713/ab.250991

**Published:** 2026-06-01

**Authors:** Hui Zhou, Shanhe Wang, Rui Su, Yuxuan Song, Xiyun Zhang, Xiaoyang Lv, Wei Sun

**Affiliations:** 1 College of Animal Science and Technology, Yangzhou University, Yangzhou, China; 2 Suzhou Stud Farm Co., Ltd., Suzhou, China; 3 College of Animal Science and Technology, Northwest A&F University, Shaanxi Yangling, China; 4 Gansu Yuansheng Agriculture & Animal Husbandry Technology Co., Ltd., Gansu, China; 5 Joint International Research Laboratory of Agriculture and Agri-Product Safety of the Ministry of Education, Yangzhou University, Yangzhou, China; 6 International Joint Research Laboratory in Universities of Jiangsu Province of China for Domestic Animal Germplasm Resources and Genetic Improvement, Yangzhou University, Yangzhou, China

**Keywords:** *BMP4*, Dermal Papilla Cells, Hu Sheep, lncBMP4, Transcriptional Regulation

## Abstract

**Objective:**

Dermal papilla cells (DPCs) are the regulatory center of hair follicle (HF) development, and their proliferation is critical in HF growth studies. Long non-coding RNAs (lncRNAs) have been demonstrated to regulate the HF growth cycle. This study aims to elucidate the molecular mechanisms by which lncRNA regulates HF formation.

**Methods:**

Based on previous RNA sequencing results, this study identified differentially expressed lncRNA-lncBMP4 in Hu sheep skin tissues with distinct patterns. The lncBMP4 was characterized by using nuclear-cytoplasmic localization and fluorescence *in situ* hybridization. Its effect on DPCs proliferation was validated using reverse transcription quantitative polymerase chain reaction, CCK-8, and EdU assays. Subsequently, we further investigated the regulatory role of lncBMP4 on *BMP4* using a Dual Luciferase Assay and an RNA immunoprecipitation experiment.

**Results:**

This study found that lncBMP4 is primarily localized in the cell nucleus and can inhibit the proliferation of Hu sheep DPCs. It acts by modulating the *BMP4* promoter activity, which influences *BMP4* expression and, consequently, the proliferation of Hu sheep DPCs.

**Conclusion:**

The current study elucidates the role and molecular mechanism of lncBMP4 in the pattern development of Hu sheep.

## INTRODUCTION

Hu sheep are a renowned breed of white lambskin in China, possessing significant economic and conservation value. Lambskin quality depends on pattern type, width, coat density, and gloss [1]. In recent years, a growing emphasis on meat production as a breeding objective has led to the neglect of the original Hu sheep skin traits. This shift has led to a decline in lambskin quality, posing a significant threat to the preservation of germplasm resources.

Dermal papilla cells (DPCs) are central regulators of hair follicle (HF) growth and development, directing epithelial cell proliferation and differentiation [2–4]. Notably, studies have established that DPCs not only induce new HF formation but also determine hair shape [5,6]. Furthermore, during the hair growth cycle, hair morphology changes in response to variations in DPC count; for example, conditional DPC knockout in mice induces a straight-to-curly hair shape transition. Building on these findings, Nissimov and Das Chaudhuri [7] proposed the “multiple dermal papilla centers” hypothesis, suggesting that not all DPCs function simultaneously, resulting in variability in the growth rates of individual follicles.

Much of the non-coding region of the human genome was once considered “junk DNA.” However, advancements in next-generation sequencing technologies have enabled the in-depth study of the non-coding genome. These non-coding RNAs participate in gene regulation through various mechanisms [8–10]. Among the different types of non-protein-coding transcripts, long non-coding RNAs (lncRNAs) have attracted increasing attention and are recognized for their diverse functions, including cis- and trans-regulatory roles, maintenance of nuclear structural integrity, and regulation of protein and RNA molecules [11–14]. In the cytoplasm, lncRNAs can act as competing endogenous RNAs (ceRNAs) for miRNAs or interact with RNA-binding proteins to modulate signaling pathways [15,16]. Additionally, short peptides generated from specific lncRNAs also exhibit regulatory functions [17,18].

LncRNAs participate in various biological processes, including embryonic development, cell differentiation, proliferation, and apoptosis. For example, Fan et al. [19] revealed key lncRNAs and genes associated with cashmere shedding in goats through transcriptome analysis. Zhu et al. [20] reported that lncRNA-H19 in secondary HFs of Liaoning cashmere goats is significantly lower in telogen and catagen than in anagen, potentially due to promoter methylation. Similarly, transcriptomic analysis of HF cycles in Angora rabbits indicated that LNC_002919 and NOVICE_CIRC_0026326 function as ceRNAs that regulate the HF cycle through miRNA-320-3p [21]. Additionally, Ge et al. [22] concluded that melatonin regulates secondary HF development in cashmere goats. Moreover, Si et al. [23] discovered that lncRNA PlncRNA-1 regulates the proliferation and differentiation of HFSCs via the TGF-β1-mediated Wnt/β-catenin signaling pathway.

Bone morphogenetic proteins (BMPs) were first found to induce bone differentiation. They prompt inducible osteogenic precursor cells (IOPCs) to become osteoblasts [24,25]. BMPs also affect HF morphogenesis, postnatal regeneration, and HF cycles by modulating DPC proliferation and differentiation [26]. Importantly, the balance between BMP2 and its inhibitor Noggin during HF affects follicle development [27]. For example, Sun et al. [28] used gene chip technology and reverse transcription quantitative polymerase chain reaction (RT-qPCR) to identify differential gene expression in the small waves of Hu sheep, suggesting that *BMP7* may influence lamb HF development. Similarly, Kulessa et al. [29] found that *BMP4* is expressed in hair shaft precursors, dermal papilla, and hair placodes in mice, suggesting a potential relationship between *BMP4* and HF development.

Although the role of *BMP4* in HF growth and development has been studied, the regulatory mechanisms remain unclear. This study identifies differentially expressed lncRNAs upstream of *BMP4* in sequencing data to reveal mechanisms of DPC proliferation from the perspective of lncRNA-mediated gene expression regulation. This approach provides new insights into the patterns of lamb pelts in Hu sheep.

## MATERIALS AND METHODS

### Experimental animals

The three 3-day-old lambs with small waves and three 3-day-old lambs with straight were selected, and the skin tissue of about 1 cm^2^ in the scapula of each lamb was carefully collected, snap-frozen in liquid nitrogen, and stored at −80°C until use.

### Cell culture and cell transfection

DPCs were cultured in DMEM/F12 (Hyclone) containing 5% fetal bovine (Gibco) serum and 1% penicillin-streptomycin (Gibco) at 37°C with 5% CO_2_. DPCs in the growth stage were inoculated uniformly in 6-well, 12-well, and 96-well plates. When the cells reached 50%–80% confluency, they were transfected with the jetPRIME reagent, following the manufacturer’s instructions. RNA and protein were extracted and stored at −80°C.

### Reverse transcription quantitative polymerase chain reaction

Total RNA from DPCs was extracted by TRIzol Universal reagent (TIANGEN), and transcribed into cDNA using the FastKing gDNA Dispelling RT SuperMix kit (TIANGEN) or the InRcute IncRNA First-Strand cDNA kit (TIANGEN), respectively. The primer information was listed in Supplement 1. RT-qPCR was performed using the 2×TSINGKE Master qPCR Mix (TSINGKE) on a CFX96 Real-Time PCR Detection System (Bio-Rad). Target gene expression levels were analyzed by the 2^−ΔΔCt^ method.

### Subcellular localization

The nuclear and cytoplasmic fractions of DPCs were isolated using the PARIS Kit (Thermo Fisher Scientific), followed by independent RNA extraction from each compartment, and gene expression was measured by RT-qPCR analysis. The U6 was used as a nuclear control, and GAPDH was used as a cytoplasmic control. The cells were processed using the Ribo Fluorescent *In Situ* Hybridization Kit (RiboBio) and the FISH Probe Mix kit (RiboBio), and the results were observed using a laser confocal microscope (ZEISS) after the cells were fixed.

### Cell proliferation assay

After the cells were transfected for 24 h, 10 μL of CCK-8 reagent (Vazyme) was added, and the cells were incubated at 37°C for 2 h. OD values at 450 nm were measured with a microplate reader (Tecan) to mark 0 h. OD measurements were taken again at 24, 48, and 72 h. In addition, cells were treated with Cell-Light EdU Apollo 567 *In vitro* Kit 48 h after transfection, and images were captured with an inverted fluorescence microscope (Nikon).

### Western blot assay

Total protein was extracted using RIPA lysis buffer, and the concentration was measured by the BCA method (Beyotime). Protein samples were separated on 10% SDS-PAGE gels (Yamei), 80 V for 20 min, then 120 V for 40 min, and transferred to PVDF membranes. After blocking with 5% sealing solution for 1 h, the membranes were incubated with primary antibody (Supplement 2) overnight at 4°C, washed 3 times with TBST for 5 min each, and incubated with secondary antibody for 1 h. Wash the PVDF membrane with TBST 3 times, 5 minutes each time, expose the bands using the ECL kit (Biosharp), and analyze the results with Image-Pro software.

### Vector construction

The coding sequence (CDS) of the Ovis aries BMP4 gene (accession number: NM_001110277.1) was obtained from the NCBI database and amplified from Hu sheep skin tissue cDNA. Next, the pcDNA3.1(+) vector was linearized by double digestion with QuickCut Hind III and QuickCut Big II (Takara). After purification, the BMP4 fragment was ligated into the linearized vector using the ClonExpress II One Step Cloning Kit (Vazyme), resulting in the recombinant pcDNA3.1(+)-BMP4 expression plasmid. Similarly, the deletion vector targeting the *BMP4* promoter region was constructed using the same procedure.

### RNA immunoprecipitation-reverse transcription quantitative polymerase chain reaction

The cells were processed according to the manufacturer’s protocol of PureBinding RNA Immunoprecipitation (RIP) Kit (GENESEEO). Immunoprecipitation was performed using specific anti-GR antibodies, with rabbit IgG as a negative control for nonspecific binding. Subsequently, Western blot analysis was performed to examine the Input, RIP, and IgG protein fractions.

### Chromatin immunoprecipitation-quantitative polymerase chain reaction

Chromatin immunoprecipitation (ChIP) assays were performed using a ChIP kit (GeneCreate). Cells cultured in 10 cm dishes were first cross-linked with 1% formaldehyde. Next, chromatin was sonicated to 200–700 bp fragments. A portion of the chromatin was saved as Input, while the remaining chromatin was immunoprecipitated with either anti-GR antibody or rabbit IgG antibody. After cross-link reversal and DNA purification, we identified putative GR-binding regions within the *BMP4* promoter and designed primers for RT-qPCR analysis.

### Dual-Luciferase reporter assay

Cells were lysed with 1 mL of 1×Cell Lysis Buffer for 10 minutes. The lysates were then transferred to 183 1.5 mL microcentrifuge tubes and centrifuged at 12,000×g for 5 minutes. Supernatant aliquots of 20 184 μL were next loaded into a 96-well black plate. followed by mixing each well with 100 μL Luciferase Substrate (Vazyme). Firefly luciferase activity was measured, then 100 μL of Renilla Substrate Working Solution was added, gently mixed to prevent bubble formation, and Renilla luciferase activity was measured. Firefly/Renilla luciferase ratios were calculated and statistically analyzed.

### Bioinformatics analysis of BMP4

The physicochemical properties of the BMP4 protein were predicted using the ProtParam tool (https://web.expasy.org/protparam/). Hydrophobicity and hydrophilicity of the BMP4 protein were predicted using ProtScale (https://web.expasy.org/protscale/). The conserved domains of the BMP4 protein were predicted using InterPro (https://www.ebi.ac.uk/interpro/). Signal peptides of the protein were predicted using SignalP 4.1 (https://services.healthtech.dtu.dk/services/SignalP-4.1/). Transmembrane regions of the protein were predicted using TMHMM software (https://services.healthtech.dtu.dk/services/TMHMM-2.0/). Potential phosphorylation sites of amino acids were predicted using the NetOGlyc 4.0 Server (NetOGlyc 4.0 Server). The secondary structure of the protein was predicted using SOPMA software (https://npsa.lyon.inserm.fr/cgi-bin/npsa_automat.pl?page=/NPSA/npsa_sopma.html). The tertiary structure of the protein was predicted using SWISS-MODEL (https://swissmodel.expasy.org/).

### Statistical analysis

Statistical analyses were performed using GraphPad Prism 9. Differences between the two groups were assessed with an independent-samples t-test, and comparisons among multiple groups were conducted using a one-way analysis of variance (ANOVA). All data are expressed as the mean±standard deviation. In figures, statistical significance is denoted as * for p<0.05 and ** for p<0.01.

## RESULTS

### Expression of lncBMP4 in skin tissues of Hu sheep

**Figure 1:**
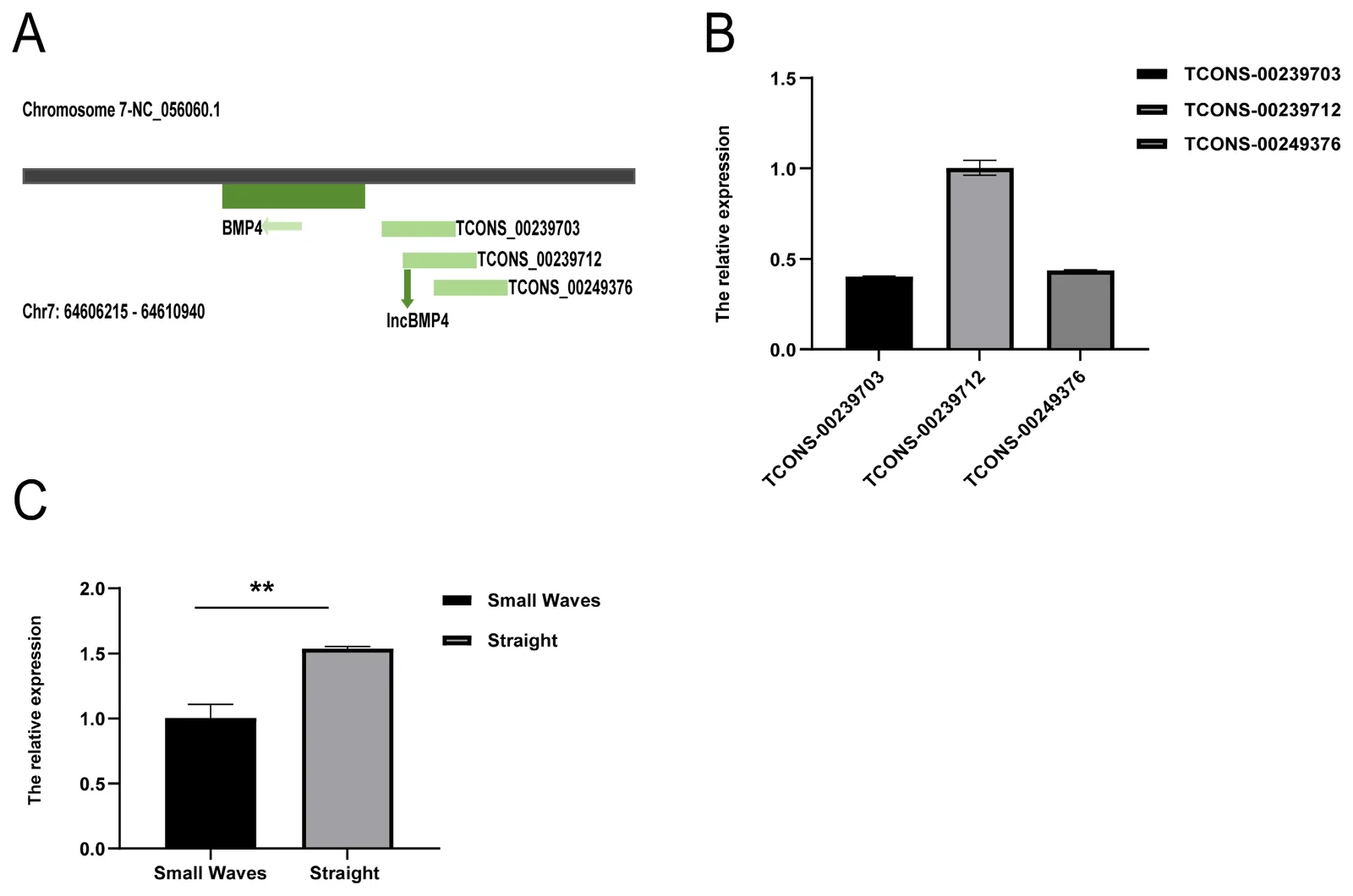
Screening of lncRNAs. (A) The location of lncBMP4. (B) Different lncRNAs’ relative expression in Hu sheep DPCs. (C) Expression levels of lncBMP4 detected by RT-qPCR in skin tissues from small waves and straight. ** p<0.01. lncRNA, long non-coding RNA; DPC, dermal papilla cell; RT-qPCR, reverse transcription quantitative polymerase chain reaction.

In the preliminary sequencing analysis, differential lncRNAs within 100 kb upstream of the BMP4 gene were identified by co-localization analysis. These included TCONS_00239703, TCONS_00239712, and TCONS_00249376 (Figure 1A). To further evaluate their significance, their potential regulatory roles were then assessed. Specifically, the expression levels of these candidate lncRNAs were examined in DPCs using RT-qPCR. TCONS_00239712 showed the highest mRNA expression among the three candidates (Figure 1B).

Further validation was performed to determine the expression pattern of TCONS_00239712 in different skin tissue types of Hu sheep. Comparative analysis demonstrated that TCONS_00239712 expression was significantly upregulated in straight compared to small waves (Figure 1C). Based on these findings, TCONS_00239712 was selected for subsequent functional studies. In accordance with lncRNA nomenclature conventions, we designated this transcript as lncBMP4.

### Subcellular localization of lncBMP4

**Figure 2:**
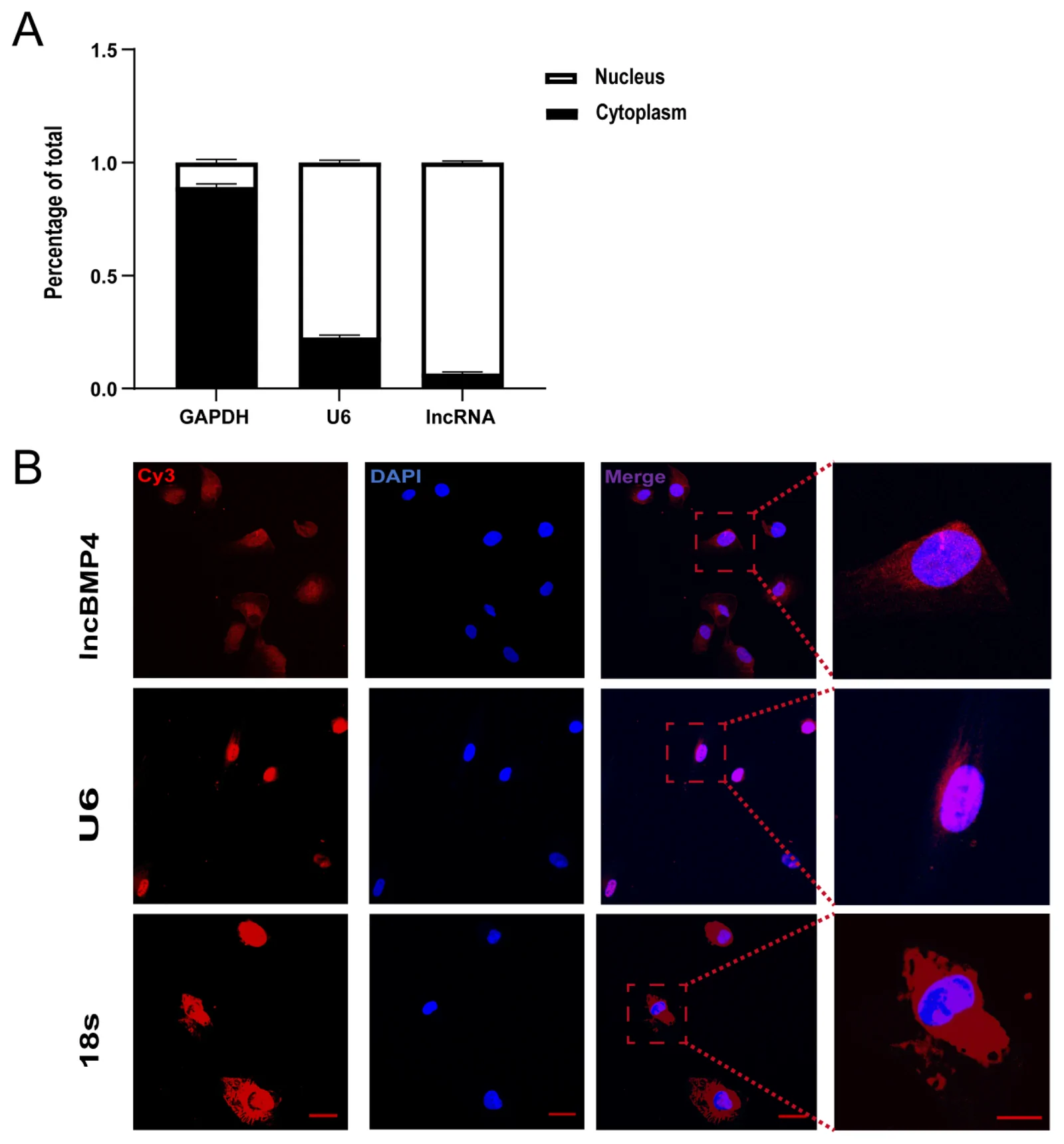
Localization of lncBMP4 in DPCs. (A) Percentage of lncBMP4 expression in the nucleus/cytoplasm of DPCs. (B) lncBMP4 FISH was performed on DPCs using Cy3-labelled probes (400×). DPC, dermal papilla cell.

We used RT-qPCR to detect lncBMP4 expression in nuclear and cytoplasmic RNA. The cytoplasmic endogenous reference gene GAPDH is expressed at about 10% expression in nuclear RNA and 90% in cytoplasmic RNA. The nuclear endogenous reference gene U6 showed about 80% expression in nuclear RNA and 20% in cytoplasmic RNA. lncBMP4 showed about 95% nuclear RNA and 5% cytoplasmic expression. This suggests that lncBMP4 is mainly expressed in the nucleus of DPCs (Figure 2A).

To further determine the cellular localization of lncBMP4, we employed FISH analysis, which revealed that U6 is uniformly distributed in the nucleus, 18S is uniformly distributed in the cytoplasm, and lncBMP4 is primarily distributed in the nucleus, consistent with the results of the Nuclear/cytoplasmic localization experiment. Therefore, we speculate that lncBMP4 may regulate the expression of downstream genes (Figure 2B).

### Effect of lncBMP4 on the proliferation of Hu sheep dermal papilla cells

**Figure 3:**
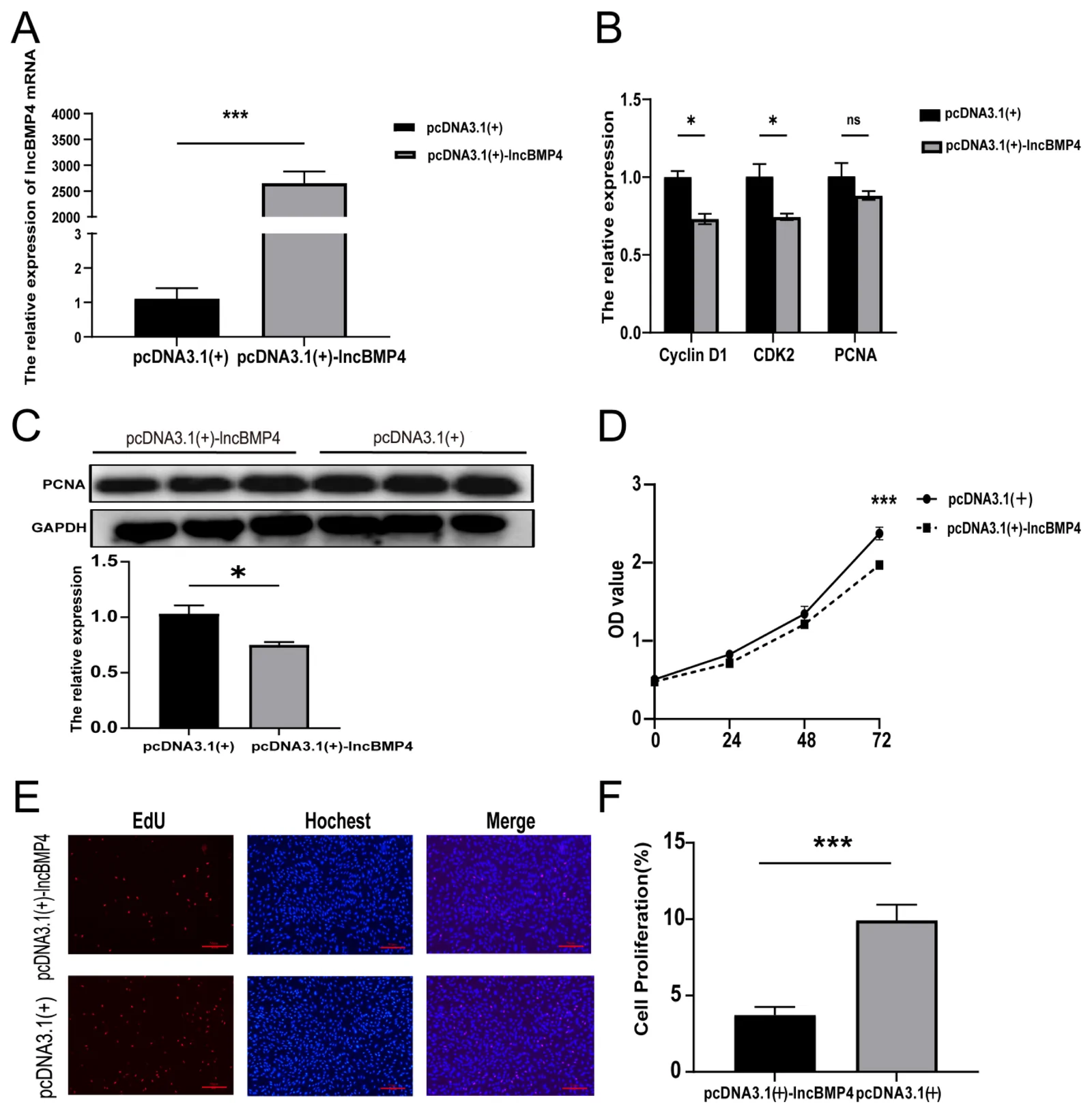
Overexpression of lncBMP4 inhibited the proliferation of Hu sheep DPCs. (A) Vector construction. (B) mRNA expression levels of *PCNA*, *Cyclin D1*, and *CDK2* after lncBMP4 overexpression. (C) The relative expression of PCNA protein after lncBMP4 overexpression. (D) Analysis of lncBMP4 on DPC proliferation at 0, 24, 48, and 72 h using the CCK-8 assay. (E) The number of proliferating Hu sheep DPCs after lncBMP4 overexpression was detected by the EdU assay. EdU staining (red) for proliferating cells. Hoechst staining (blue) for the nucleus. (F) The proportion of EdU-positive Hu sheep DPCs. * p<0.05, ** p<0.01, *** p<0.001. DPC, dermal papilla cell.

First, we successfully constructed the pcDNA3.1(+)-lncBMP4 vector (Figure 3A). Next, RT-qPCR results showed that the relative expression levels of *CDK2* and *Cyclin D1* mRNA were significantly reduced after overexpression of lncBMP4. However, *PCNA* mRNA levels did not change significantly (Figure 3B). In contrast, western blot results showed that PCNA protein levels significantly decreased after overexpression of lncBMP4 (Figure 3C). Furthermore, we used the CCK-8 assay to analyze the effect of lncBMP4 on DPC proliferation. After lncBMP4 overexpression, cell viability in the pcDNA3.1(+)-lncBMP4 group was significantly reduced at 72 h compared to the pcDNA3.1(+) group (Figure 3D). The EdU assay also showed that the number of cells in the pcDNA3.1(+)-lncBMP4 group during the proliferation phase was significantly lower than that in the pcDNA3.1(+) group (Figures 3E, 3F). The results showed that lncBMP4 inhibited the proliferation of Hu sheep DPCs.

### lncBMP4 regulates the promoter activity of the BMP4 gene

**Figure 4:**
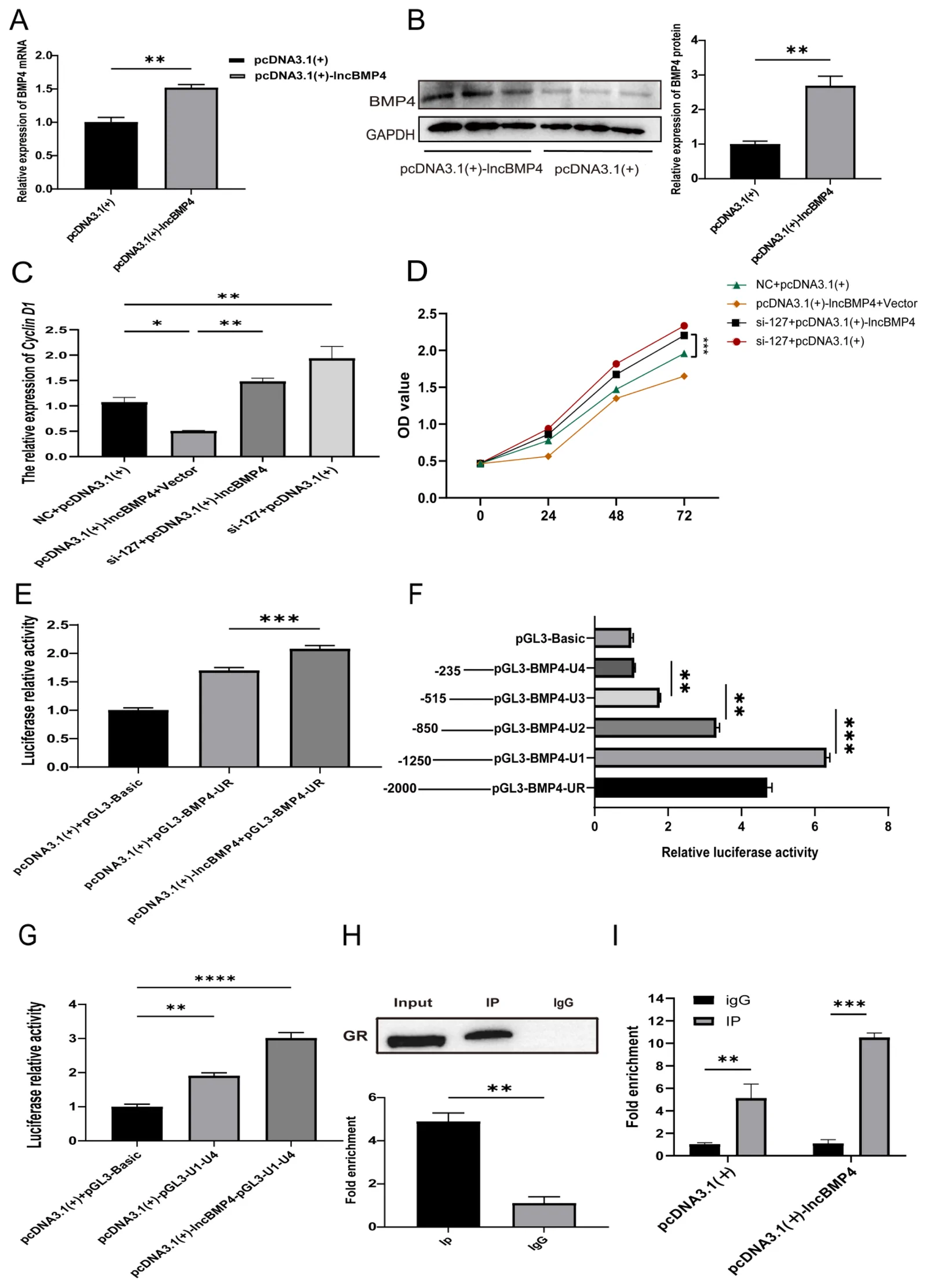
lncBMP4 transcriptionally regulates the expression of *BMP4*. (A) The relative expression levels of *BMP4* mRNA after lncBMP4 overexpression. (B) The relative expression levels of BMP4 protein after lncBMP4 overexpression. (C) mRNA expression levels of *PCNA*, *Cyclin D1*, and *CDK2* in the rescue experiment. (D) Analysis of the rescue experiment on DPC proliferation at 0, 24, 48, and 72 h using the CCK-8 assay. (E) Effect of lncBMP4 overexpression on the *BMP4* promoter activity. (F) Detection of dual luciferase activity in different regions of the *BMP4* promoter region. (G) Effect of lncBMP4 overexpression on *BMP4* core promoter activity. (H) Result of RIP assay. (I) Result of ChIP assay. * p<0.05, ** p<0.01, *** p<0.001, **** p<0.0001. DPC, dermal papilla cell; ChIP, chromatin immunoprecipitation.

The RT-qPCR results showed that lncBMP4 overexpression in DPCs significantly increased BMP4 mRNA expression (Figure 4A). Consistently, Western blot analysis confirmed this effect at the protein level, showing a statistically significant increase in BMP4 protein abundance after lncBMP4 overexpression (Figure 4B). To further verify whether lncBMP4 mediates BMP4 activity that affects DPC proliferation, we performed co-transfection with lncBMP4 overexpression and si-127. The mRNA expression levels of the related genes in the co-transfection group were significantly higher than those in the lncBMP4 overexpression group (Figure 4C). Additionally, at 72 hours, the CCK-8 assay showed the OD value was significantly higher in the co-transfection group (Figure 4D).

To test whether lncBMP4 overexpression affects *BMP4* promoter activity, we constructed the pGL3-BMP4-UR vector. The pcDNA3.1(+)-lncBMP4 and pcDNA3.1(+) vectors were co-transfected into 293T cells with the pGL3-BMP4-UR vector, respectively, using pRL-TK as an internal reference. A Dual luciferase assay showed a significant increase in *BMP4* promoter activity following lncBMP4 overexpression (Figure 4E).

The pGL3-Basic vector and the vector with different deletions in the promoter region of the BMP4 gene were co-transfected with the pRL-TK plasmid into 293T cells. A Dual luciferase assay showed that promoter activity decreased significantly when truncated from −1,250 bp to −850 bp, from −850 bp to −515 bp, and from −515 bp to −235 bp. Thus, the region from −1,250 bp to −235 bp is the *BMP4* core promoter (Figure 4F).

The pGL3-U1-U4 vector was designed and constructed to further test whether lncBMP4 affects the activity of the *BMP4* core promoter. The results of the dual luciferase assay showed that overexpression of lncBMP4 significantly increased the activity of the *BMP4* core promoter (Figure 4G).

### Prediction and validation of transcription factors in the core promoter region of the BMP4 gene in Hu sheep

The transcription factors (TFs) within the core promoter region of the BMP4 gene were predicted utilizing the AnimalTFDB4.0 and Alibaba2.1 databases. The results demonstrated that GR, SP1, and GATA-1 were consistently identified by both prediction tools (Supplement 3).

To evaluate binding between lncBMP4 and predicted TFs (GR, SP1, GATA-1), we used the catRAPID platform (Supplement 4). GR was chosen for further study. RIP assay showed that GR was significantly enriched at lncBMP4, supporting a direct interaction between lncBMP4 and GR (Figure 4H). ChIP assays revealed that lncBMP4 overexpression significantly increased the enrichment of GR at the *BMP4* promoter (Figure 4I).

### Bioinformatics analysis of BMP4 in Hu sheep

**Figure 5:**
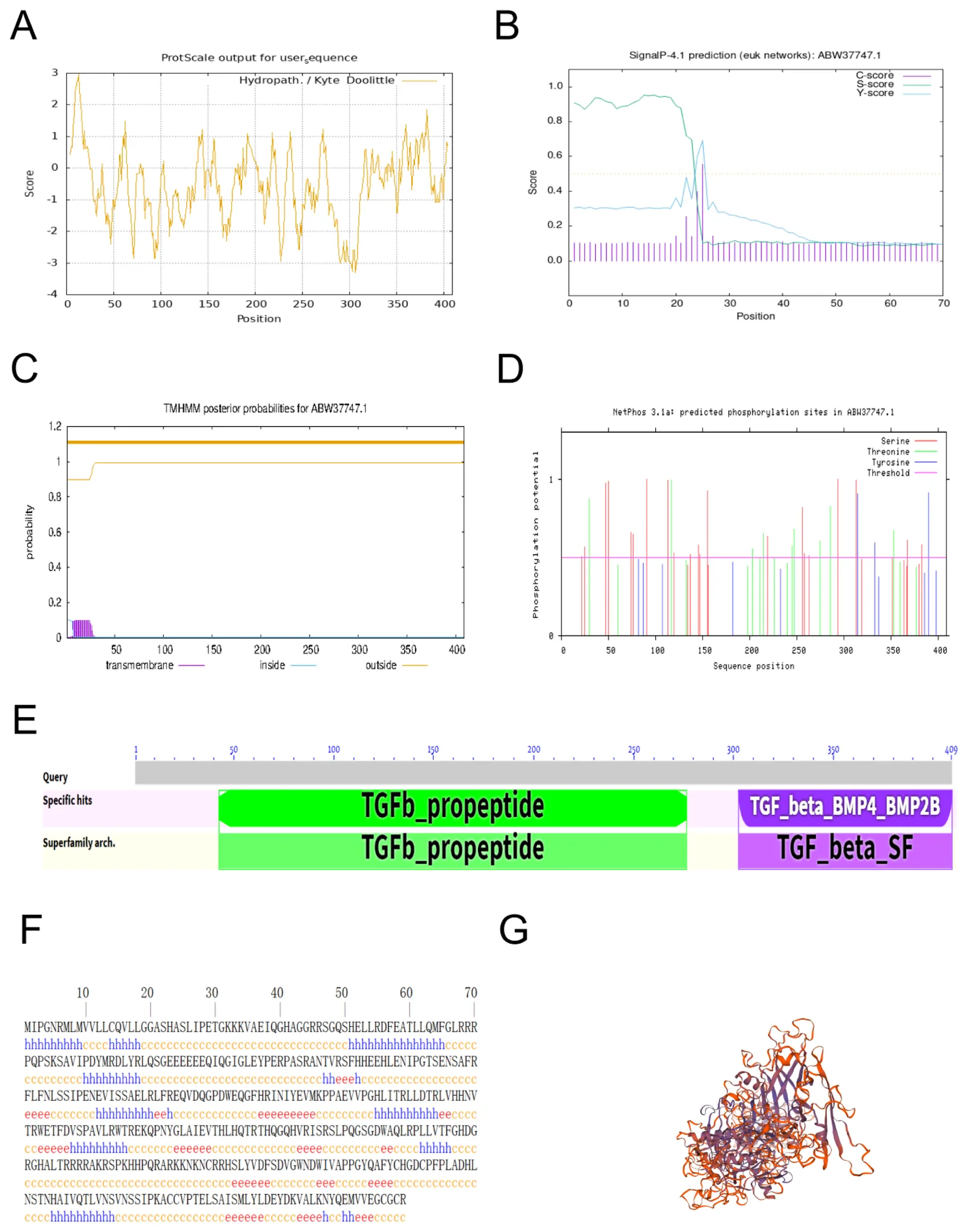
Bioinformatics analysis of *BMP4* in Hu sheep. (A) BMP4 protein hydrophobicity analysis. (B) BMP4 signal peptide prediction. (C) BMP4 transmembrane structure prediction. (D) Predicted potential phosphorylation sites of the BMP4 protein. (E) Predicted structural domain of the BMP4 protein. (F) Secondary structure analysis of the BMP4 protein. (G) Predicted tertiary structure of BMP4 protein.

To systematically characterize BMP4, we performed computational analyses. ProtParam revealed that BMP4 is a 409-amino acid polypeptide with a high leucine content and low tryptophan content. It contains 50 positively charged and 46 negatively charged residues. A GRAVY score of −0.549 indicates overall hydrophilicity. ProtScale analysis confirmed strong hydrophilicity, with lysine 307 being the most hydrophilic and cysteine 14 the most hydrophobic (Figure 5A). SignalP4.1 predicted a signal peptide (Figure 5B). TMHMM indicated no transmembrane region, supporting secretion and possible membrane-receptor binding (Figure 5C). NetPhos 3.1 predicted 33 phosphorylation sites (Figure 5D). CDD analysis revealed a TGF-β peptide and a TGF-β domain, suggesting involvement in TGF-β signaling (Figure 5E). SOPMA predicted a secondary structure dominated by random coils with α-helices, extended strands, and β-turns (Figure 5F). Homology modeling indicated a tertiary structure composed mainly of random coils, α-helices, and β-turns (Figure 5G).

### BMP4 inhibits the proliferation of dermal papilla cells in Hu sheep

**Figure 6:**
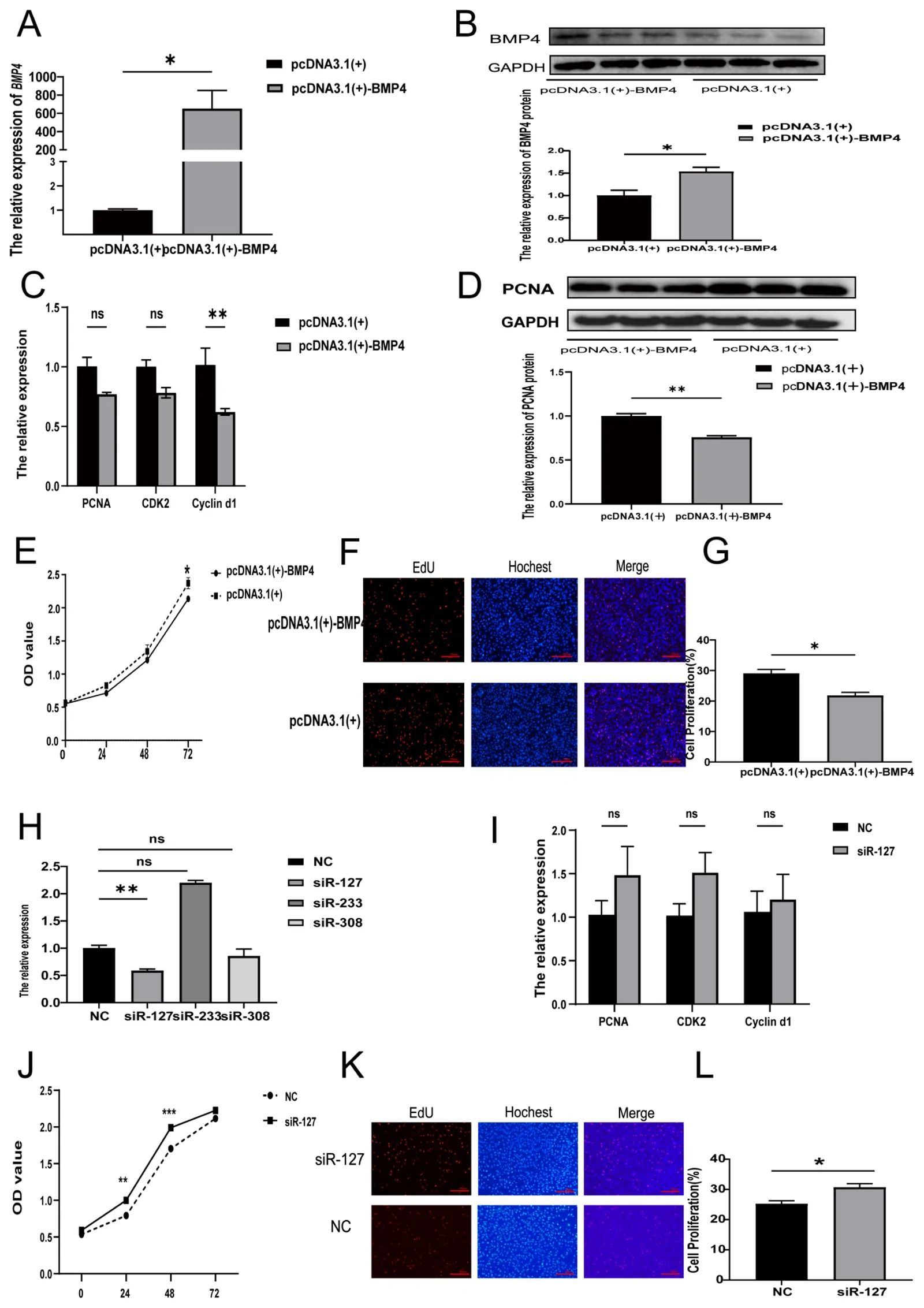
Effect of *BMP4* on the proliferation of Hu sheep DPCs. (A) The expression levels of *BMP4* mRNA after *BMP4* overexpression. (B) The expression levels of *BMP4* protein after *BMP4* overexpression. (C) The relative expression levels of *PCNA*, *Cyclin D1*, and *CDK2* mRNA after *BMP4* overexpression. (D) The relative expression levels of PCNA protein after *BMP4* overexpression. (E) Analysis of the effect of *BMP4* overexpression on DPC proliferation at 0, 24, 48, and 72 h using the CCK-8 assay. (F) The proliferation of DPCs after overexpression of BMP4 by EdU assay (100×). EdU staining (red) for proliferating cells. Hoechst staining (blue) for the nucleus. (G) The proportion of EdU-positive Hu sheep DPCs. (H) The relative expression of *BMP4* mRNA after *BMP4* knockdown. (I) The relative expression levels of *PCNA*, *Cyclin D1*, and *CDK2* mRNA after *BMP4* knockdown. (J) Analysis of the effect of *BMP4* knockdown on DPC proliferation at 0, 24, 48, and 72 h using the CCK-8 assay. (K) The proliferation of DPCs after *BMP4* knockdown by EdU assay (100×). EdU staining (red) for proliferating cells. Hoechst staining (blue) for the nucleus. (L) The proportion of EdU positive Hu sheep DPCs. * p<0.05, ** p<0.01, *** p<0.001. DPC, dermal papilla cell.

We constructed a pcDNA3.1(+)-BMP4 vector, and RT-qPCR confirmed that *BMP4* overexpression significantly increased its mRNA (Figure 6A). The Western blot result further showed elevated BMP4 protein levels (Figure 6B). RT-qPCR results showed that following BMP4 overexpression, the expression levels of the proliferation-related genes *PCNA* and *CDK2* exhibited a downward trend, while *Cyclin D1* expression decreased significantly. Western blot analysis confirmed that PCNA protein levels decreased significantly after *BMP4* overexpression. The CCK-8 assay showed that the cell viability of the treatment group was significantly lower than that of the control group at 72 h (Figure 6E). In addition, the EdU assay showed that the number of EdU-positive cells was significantly decreased (Figures 6F, 6G). The results above indicate that *BMP4* overexpression can inhibit DPC proliferation. Among the three knockdown vectors, the RT-qPCR confirmed that siRNA-127 significantly reduced *BMP4* mRNA levels (Figure 6H). Moreover, siRNA-127 upregulated mRNA expression of *PCNA*, *CDK2*, and *Cyclin 305 D1* (Figure 6I).. Furthermore, CCK-8 assays showed significantly higher cell viability in the siRNA-127 group than in the NC group at 24 and 48 h (Figure 6J). Similarly, EdU assays demonstrated a significantly higher proportion of proliferating cells following *BMP4* knockdown (Figures 6K, 6L). These results further demonstrate that *BMP4* can inhibit the proliferation of PDCs.

## DISCUSSION

Currently, lncRNAs are thought to play important roles in many biological processes, including the cell cycle, differentiation, metabolism, and disease [30–32]. The cellular location of RNA may determine whether it is stored, processed, translated, or degraded [33,34]. Guo et al. [35] demonstrated that FAST is predominantly localized in the cytoplasm of human embryonic stem cells (hESCs). It interacts with the WD40 domain of the E3 ubiquitin ligase b-TrCP, inhibiting the interaction of phosphorylated β-catenin with and preventing its degradation, thereby activating the WNT signaling pathway. In contrast, FAST is localised to the nucleus in mouse embryonic stem cells (mESCs), but its processing is inhibited by the splicing factor PPIE. These results suggest that localised processing of lncRNAs is tightly linked to function. In this study, we identified a differentially expressed lncRNA located 100 kb upstream of *BMP4* in Hu sheep. Using nuclear-cytoplasmic separation and fluorescence *in situ* hybridization, we determined that lncBMP4 is primarily localized in the nuclei of DPCs. It has been shown that nuclear-localised lncRNAs not only interact with chromatin but also regulate gene transcription. For example, Simion et al. [36] reported that lncRNA MAARS binds HuR in the nucleus to regulate its targets, contributing to atherosclerosis. In addition, nuclear-localised lncRNAs can regulate variable splicing of mRNAs. Huan et al. [37] demonstrated that nuclear LUCAT1 interacts with PTBP1 to alter the splicing of DNA damage-related genes, promoting colorectal cancer progression and drug resistance.

There are currently numerous studies on the role of lncRNAs in HF growth and development. Jiao et al. [38] investigated the transcriptional pattern of lncRNAs in secondary HFs of the velvet goat and constructed a related ceRNA regulatory network. The results showed that 4 of the 16 differentially expressed lncRNAs screened were significantly up-regulated, and 9 were significantly down-regulated. In this study, lncBMP4, localised in the nucleus, was functionally verified and found to inhibit DPC proliferation.

LncRNA expression is regulated at the epigenetic, transcriptional, and post-transcriptional levels. For example, Xiang et al. [39] identified CCAT1-L, a lncRNA located upstream of *MYC*, which functions in the transcriptional regulation of MYC and promotes the formation of distal chromatin stem-loop structures; Lee and Bartolomei [40] reported that some lncRNA promoters bind to differentially methylated regions (DMRs), and knockdown of these promoters resulted in the loss of imprinting of protein-coding genes. A common regulatory mechanism is post-transcriptional regulation, in which lncRNAs modulate or act as miRNA sponges. Exactly single-cell sequencing data, Qiu et al. [41] found that RNA editing is more likely to occur at RNA splice sites during early human embryonic development. In a notable study, Ørom et al. [42] identified lncRNAs as enhancers, demonstrating that knockdown of specific lncRNAs decreased the expression of neighboring protein-coding genes, including TAL1, Snai1, and Snai2. Similarly, Cai et al. [43] lncRNA-Six1, located upstream of the *Six1*, showed that its overexpression enhances both mRNA and protein levels of the *Six1*. Conversely, the knockdown of lncRNA-Six1 represses *Six1* expression, indicating that lncRNA-Six1 functions in cis-regulation of the *Six1*.

In this study, lncBMP4 overexpression significantly increased *BMP4* mRNA and protein levels. To further elucidate the underlying mechanism, we constructed *BMP4* promoter vectors and found that lncBMP4 overexpression enhanced promoter activity. The software predicted TFs associated with the *BMP4* promoter region, including *GR*, *SP1*, and *GATA-1*. Among these candidates, we selected GR because it is associated with HF development and growth [44]. The RIP assay confirmed the interaction between lncBMP4 and GR, and the ChIP assay showed that lncBMP4 overexpression significantly increased GR enrichment at the *BMP4* promoter.

These results demonstrate that lncBMP4 can regulate the transcriptional activity of the *BMP4* promoter. Wang et al. [45] discovered a significant negative correlation between *BMP4* expression and HF density in Akan fine-wool sheep, suggesting that *BMP4* may suppress HF density. In functional validation experiments, we found that *BMP4* inhibits DPC proliferation. Previous studies have shown that the number and proliferative activity of DPCs are critical determinants of hair curvature [2,6]. Our study demonstrates that *BMP4* inhibits the proliferation of Hu sheep DPCs, suggesting that *BMP4* may influence hair curvature by negatively regulating these cells. These findings provide a foundation for further mechanistic studies.

## CONCLUSION

In summary, this study found that lncBMP4 is mainly localised in the nucleus and can inhibit the proliferation of Hu sheep DPCs, and can influence the expression of *BMP4* by mediating the promoter activity of the *BMP4*, which in turn affects the proliferation of the DPCs, and provides a theoretical basis for further study of the mechanism of Hu sheep lambskin pattern.

## SUPPLEMENTARY MATERIAL

**Supplement 1.** The Primer sequences of genes used for RT-qPCR in this experiment.

**Supplement 2.** The primary antibody and secondary antibody information.

**Supplement 3.** Prediction of transcription factors of *BMP4* core promoter in Hu sheep.

**Supplement 4.** Predicting the binding capacity between lncBMP4 and transcription factors.
