# Supplementary material for: The mechanisms of lncBMP4 mediating BMP4 promoter activity to regulate the proliferation of dermal papilla cells in Hu sheep
Source: Anim Biosci. 2026 Jun 1;39(9):250991. doi: 10.5713/ab.250991 (PMC13547618; doi:10.5713/ab.250991)
Supplement: Supplementary file 1 [file ab-250991-Supplementary-1.pdf]

Supplement 1. The Primer sequences of genes used for RT-qPCR in this experiment

Genes	Primer sequences	GenBank accession
TCONS_00239703	F: CCTCCTTCCCACCCTTATCC<br>R: AAGCCTCTTTGACCAGTCGT	
TCONS_00239712	F: ATTCCACGGACAGAAGAGCC<br>R: ATGCTCAGTCGTGTCCAACT	
TCONS_00249376	F: CTGTGTGGTCAAGCATCGAG<br>R: TCTGAAGGATTTGCGCGTGA	
CDK2	F: AGAAGTGGCTGCATCACAAG<br>R: TCTCAGAATCTCCAGGGAATAG	XM_012158800.5
PCNA	F: CGAGGGCTTCGACACTTAC<br>R: GTCTTCATTGCCAGCACATT	XM_004014340.5
Cyclin D1	F: CCGAGGAGAACAAGCAGATC<br>R: GAGGGTGGGTTGGAAATG	XM_027959928.2
GAPDH	F: TCTCAAGGGCATTCTAGGCTAC<br>R: GCCGAATTCATTGTCGTACCAG	NM_001190390.1
BMP4	F: CCGTGAGGAGCTTCCACCA<br>R: GTTTATACGATGAAAGCCCTGCTCC	NM_001110277.1
U6	F: CTCGCTTCGGCAGCACA<br>R: AACGCTTCACGAATTTGCGT	XM_004007918.4
GR	F: AGATGCCAAGGGTCTGGAG<br>R: AATTGTGCTGTCCTTCCACT	XM_060415326.1
ChIP-qPCR	F: AGAATTGCGTGGGTCAGGATT<br>R: CTCAGCACAGGGGCCTTAAAA	NM_001110277.1
