# Supplementary material for: The mechanisms of lncBMP4 mediating BMP4 promoter activity to regulate the proliferation of dermal papilla cells in Hu sheep
Source: Anim Biosci. 2026 Jun 1;39(9):250991. doi: 10.5713/ab.250991 (PMC13547618; doi:10.5713/ab.250991)
Supplement: Supplementary file 2 [file ab-250991-Supplementary-2.pdf]

Supplement 2. The primary antibody and secondary antibody information

Target molecule	Purpose	Origin	Dilution	Company
GAPDH	primary antibody	Mouse	2500	Proteintech
BMP4	primary antibody	Rabbit	5000	Bioss
GR	primary antibody	Rabbit	10000	Abclonal
HRP Goat Anti-Rabbit	Secondary antibody	Rabbit	10000	Abclonal
HRP Goat Anti-Rabbit	Secondary antibody	Mouse	10000	Abclonal
