# Supplementary material for: The mechanisms of lncBMP4 mediating BMP4 promoter activity to regulate the proliferation of dermal papilla cells in Hu sheep
Source: Anim Biosci. 2026 Jun 1;39(9):250991. doi: 10.5713/ab.250991 (PMC13547618; doi:10.5713/ab.250991)
Supplement: Supplementary file 3 [file ab-250991-Supplementary-3.pdf]

Supplement 3. Prediction of transcription factors of *BMP*4 core promoter in Hu sheep

Software	Transcription factor
AnimalTFDB 4.0	C/EBPalpha、GR、TCF-2、NERF-1a、AP-2、GATA-3、NF-1、STAT5A、IRF-2、STAT6、SP1、STAT1beta、Myod、HOXD8、SOX2、STAT4、Zen-1、HNF-3、BTEB4、RF2a、E2F-2、HOXA3、RAR-beta、Smad4、GATA-1、TCF-4E、EGR-1、AT-BP1、Pax-5、Oct-B1
Alibaba 2.1	RXR-beta、SpI、NF-1、HSE-bind、Oct-1、Zen-1、Ftz、GR、IRF-1、GATA-1、C/EBPalpha、REV-ErbA、ARP-1、HOXD4、TBP、MIG-1、Pit-1a、ICSBP、NF-kappa、NF-kappaB、RAP1
