# Supplementary material for: The mechanisms of lncBMP4 mediating BMP4 promoter activity to regulate the proliferation of dermal papilla cells in Hu sheep
Source: Anim Biosci. 2026 Jun 1;39(9):250991. doi: 10.5713/ab.250991 (PMC13547618; doi:10.5713/ab.250991)
Supplement: Supplementary file 4 [file ab-250991-Supplementary-4.pdf]

Supplement 4. Predicting the binding capacity between lncBMP4 and transcription factors

Protein	RNA	Interaction propensity	Z-value	RBP propensity	RNA-binding domains
GR		18.32	0.56	1	3
Sp1	lncBMP4	21.44	0.78	0.47	3
GATA-1		7.72	0.16	0.8	2
